# The effect of hand and foot exercises on peripheral neuropathy and quality of life in women with breast cancer: a randomized controlled trial

**DOI:** 10.1007/s00520-025-09145-x

**Published:** 2025-01-08

**Authors:** Neşe Uysal, Filiz Ünal Toprak

**Affiliations:** 1 Nursing Department, Amasya University Faculty of Health Science, İpekköy, Amasya, Turkey; 2 Midwifery Department, Gülhane Faculty of Health Science, University of Health Sciences, Ankara, Turkey

**Keywords:** Breast cancer, Hand and foot exercise, Peripheral neuropathy, Quality of life

## Abstract

**Purpose:**

Peripheral neuropathy is one of the most devastating symptoms experienced by the patients. Supportive and holistic care interventions are crucial to help these patients. The aim of this study is to determine the effects of hand and foot exercises on chemotherapy-induced peripheral neuropathy and quality of life in women with breast cancer.

**Methods:**

The sample of this randomized controlled trial study consisted of 79 women with breast cancer who underwent taxane-group chemotherapy in a hospital. The women diagnosed with grade 1 or higher peripheral neuropathy were included in the study. Women were divided into three groups as exercises with a massage ball, exercises with a stress ball, and control group. Hand and foot exercises last for 8 weeks. Data were collected using the Information Form, the Common Terminology Criteria for Adverse Events, and the European Organization for the Research and Treatment of Cancer Quality of Life Questionnaire.

**Results:**

The severity of neuropathy decreased significantly in both massage ball and stress ball exercise groups compared to the control group (*p* < 0.05). The group and time interaction was statistically significant in fatigue, pain, and motor symptoms (*p* < 0.05).

**Conclusion:**

Women with breast cancer who undergo neurotoxic chemotherapy are thought to alleviate neuropathy symptoms and enhance their quality of life through simple home-based exercises. Nonpharmacological, applicable interventions, such as hand-foot exercises, can be integrated into patient education and care practices during the chemotherapy process.

**Trial registration:**

*ClinicalTrails.gov* (Registration number: NCT06055088. registered on 01 June 2023).

## Introduction

Breast cancer is one of the most prevalent cancer types that cause death in women in the world and Türkiye [1]. Chemotherapy is one of the most common treatment options in the treatment of breast cancer. However, patients with breast cancer suffer from numerous side effects due to the chemotherapy regimen used. One of the major side effects associated with the administration of taxane-based chemotherapy drugs, which are frequently used to treat breast cancer, is chemotherapy-induced peripheral neuropathy (CIPN) [2–4]. Due to their neurotoxic effects, taxane-based chemotherapy drugs can damage peripheral sensory, motor, and autonomic nerves. Patients with CIPN experience symptoms such as paresthesia, numbness, tingling, and pain in the extremities since the sensory nerve is affected. Patients with motor nerve involvement experience muscle weakness, atrophy, weakness in the muscles of the hands and feet, and impairment in fine motor movements (buttoning up, pressing buttons, grasping objects, writing, holding a pen, etc.). Autonomic symptoms include constipation or diarrhea, sweating abnormalities, and dizziness [2–4]. These CIPN-related symptoms negatively affect patients’ physical function and quality of life, and chronic pain and sensorimotor deficits raise their risk of falls and morbidity [3–5].

Symptom management in individuals undergoing chemotherapy is among the primary responsibilities of healthcare professionals. Evidence-based guidelines recommend the use of nonpharmacological approaches with proven efficacy for symptom management [6]. Exercise, one of these approaches, is easy to use, is affordable, and has no significant side effects. Exercises serve as an alternative treatment to enhance the quality of life of people with neuropathy by increasing activities of daily living and relieving pain [7, 8]. In a meta-analysis evaluating the effect of exercise on neuropathy, a nerve gliding exercise and a sensorimotor-based exercise were found to improve CIPN symptoms [9]. In other studies, it was determined that various types of exercises such as aerobic exercise, balance exercise, strength training, endurance training, nerve gliding exercises, and vibration training were applied, and it was stated that combined exercise could be an effective option to improve quality of life, physical function, and neuropathic pain [10, 11]. Despite the studies showing the beneficial effects of exercise in the literature, it was found that there were differences in terms of exercise type, frequency, and duration in exercise interventions. However, it was determined that there were a limited number of studies evaluating the effect of exercise applied specifically to the hands/feet [12, 13].

Simple, home-based exercises are advantageous since they can be made in a short period of time on a daily basis and do not require special equipment. It has been reported that simple hand and foot exercises improve hand strength and increase joint range of motion and performance [7, 8, 14]. In studies conducted with cancer patients, hand and foot exercises have been found to reduce pain intensity, reduce peripheral neuropathy symptoms, and improve daily living activities [12, 13]. Doing these exercises with various tools such as foam rollers, sticks, and massage balls brings additional benefits by creating a self-massage effect [15, 16]. Using a stress ball during exercise provides stimulation by applying pressure to many muscles and nerves in and around the hand, regulating the functioning of the nervous system and reducing the release of stress hormones, while also providing relaxation and relief [17, 18]. In exercises performed with a massage ball or similar devices, the massage effect on the muscles and the pressure effect on the tissue are used [16]. Exercises made with a tool (e.g., a foam roller, a roller massage stick, or a tennis ball) provide some of the benefits of massage, increase the range of motion, and alleviate delayed-onset muscle pain [16, 19]. Several mechanisms explain the beneficial effects of exercise on CIPN symptoms. Inflammation is involved in the etiology of CIPN and exercise reduces inflammation. Exercise changes how sensations from the hands and feet are processed by the brain (especially the thalamus, sensory-motor cortex, and insula). Exercise can eliminate the central sensitization associated with neuropathic pain and relieve CIPN-related pain [15, 16, 19, 20]. Exercise improves muscle strength and balance and reduces the progression of CIPN symptoms. Accordingly, it is important to assess the feasibility and effects of an exercise program in patients with CIPN.

Due to the increasing prevalence of cancer, the expansion of chemotherapy indications, and the lack of adequate therapeutic or preventive strategies for the side effects of chemotherapeutic agents with neurotoxic side effects such as neuropathy, new evaluation and care strategies for symptom management are gaining importance. It is important to manage side effects that negatively affect the treatment continuance to get the full benefits of chemotherapy. In this regard, oncology nurses play a key role in detecting symptoms at an early stage with multidisciplinary collaboration by observing neuropathy and its impact on life [21]. A literature review using the keywords of chemotherapy, peripheral neuropathy, and breast cancer has shown that although there are studies that evaluated the effects of neuropathic pain and neuropathy on quality of life and activities of daily living, there are a limited number of intervention studies to alleviate chemotherapy-induced neuropathy [10–13, 22, 23]. Given the lack of adequate therapeutic or preventive strategies against CIPN, new assessment and care strategies for symptom management are important. Given the long-term and adverse effects of CIPN, supportive care strategies and further studies are required to manage the burden of CIPN [4, 24].

The aim of this study was to evaluate the effect of hand-foot exercises to alleviate CIPN in women with breast cancer undergoing taxane-based chemotherapy. The secondary aim of the study is to determine whether or not exercise with a massage ball and exercise with a stress ball were superior to each other.

## Methods

### Study design

This is a prospective, randomized, controlled, experimental study with three groups. This study was conducted in accordance with Consolidated Standards of Reporting Trials (CONSORT) 2010 guidelines.

### Participants

The sample of the study consisted of women with breast cancer who underwent chemotherapy in the Gülhane Training and Research Hospital’s Outpatient Chemotherapy Unit between June 2023 and December 2023. The sample size was calculated using the G Power (v3.1.7) program. Based on the study by Sacid and Arıkan, it was determined that a total of 75 women should be reached with a type 1 error probability of 0.05%, a power of 80%, and an effect size of 0.25, according to the calculation made using quality of life scores, one of the main outputs of the study [6]. However, given the possibility of 10% data loss, it was planned to include a total of 84 women in the sample: 28 in the massage ball group, 28 in the stress ball group, and 28 in the control group.

The inclusion criteria were determined as follows: being aged between 18 and 80 years, being diagnosed with breast cancer, being able to read and speak Turkish, undergoing adjuvant or neoadjuvant taxane-based chemotherapy, having at least grade 1 neuropathy according to CTCAE, and agreeing to participate in the study. The exclusion criteria were determined as follows: having peripheral neuropathy not induced by chemotherapy (tumor compression, nutrition disorders, infections, stroke, diabetes, etc.), skin infection, scar tissue, inflammation or cuts only on the hands or feet, having undergone taxane-based chemotherapy, and having a comorbidity that can affect the sensory function of the nervous system, such as a neuropsychiatric disease.

### Randomization and blinding

**Fig. 1:**
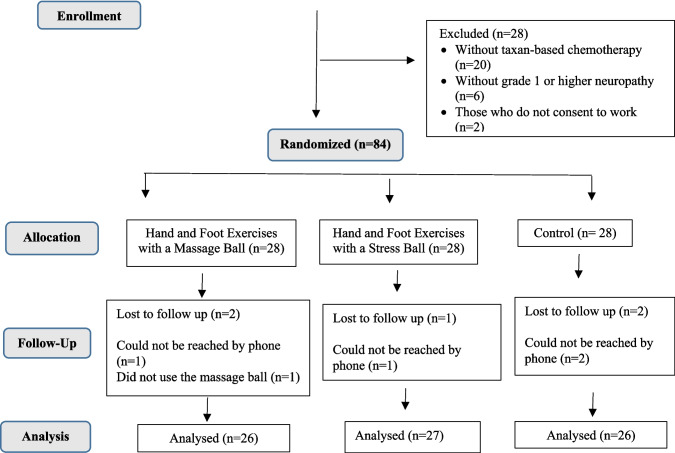
Consort chart of the study

The researcher generated a random assignment list using the randomization tool (http://www.randomizer.org) to allocate the sampled population into two intervention groups and one control group. Women with breast cancer were divided into three groups (exercises with a massage ball, exercises with a stress ball, and control groups) using the block randomization method (Fig. 1). Due to the nature of the intervention, it was not possible to blind participants or researchers.

### Intervention and data collection

The women undergoing taxane-based chemotherapy were evaluated for neuropathy during chemotherapy, and those diagnosed with grade 1 or higher neuropathy were included in the study. Based on National Cancer Institute Common Terminology Criteria for Adverse Events version 5.0 (NCI-CTCAE v5.0) criteria, symptoms such as paresthesia, presence of subjective weakness, sensory loss, and their level of limiting activities of daily living and self-care were evaluated by the researcher.

#### Exercise group (hand and foot exercises with a massage ball or a stress ball)

Home-based hand and foot exercises lasting for 8 weeks consisted of sensory exercises and strengthening exercises. The literature was reviewed for determining what the hand-foot exercises would be [4, 7, 25]. The exercise program consisted of simple, home-based exercises. The opinion of an expert physiotherapist was obtained for the exercise program. By combining the evidence from the literature and the opinions of the physiotherapist, exercise steps were formed. Women in the massage ball group used a massage ball during the exercises, while those in the stress ball group used the stress ball during the exercises.

##### Hand exercises

**Fig. 2:**
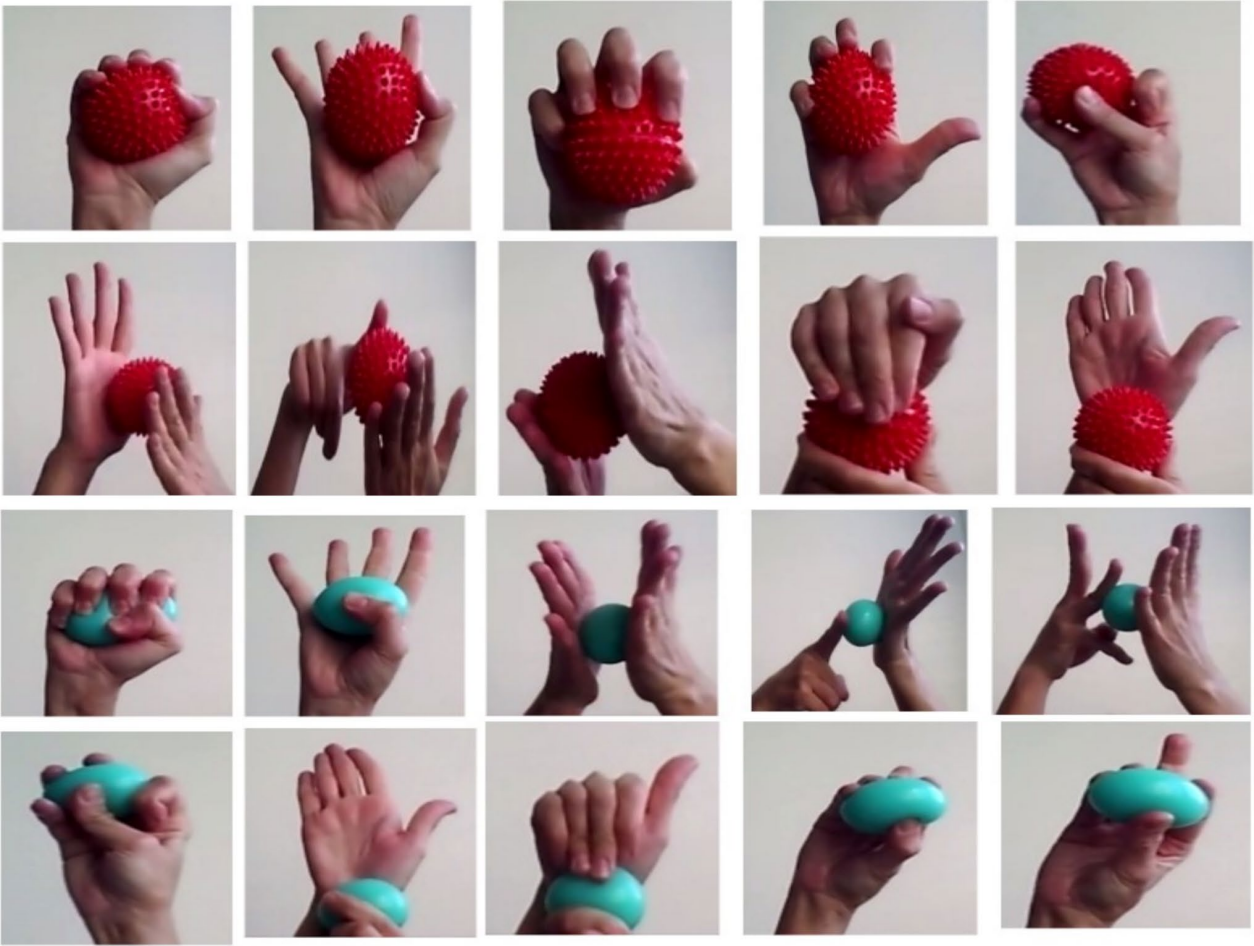
Hand and foot exercise with massage ball and stress ball

The patients were asked to place a ball between their two hands and roll it to produce light pressure during the sensory exercises and to squeeze and unclench the ball in their palm for 10 s during the strengthening exercises. The participants did movements such as stretching the wrist, tapping the fingers, circling and tensing the fingers, rolling the fingers, and circling and twiddling the thumbs for the hands (Fig. 2).

##### Foot exercises

The movements involved bending and stretching the toes slowly and tightly, lifting and tapping the toes off the floor while keeping the heels on the floor, pointing the toes out and putting them back on the floor, forming a “V” with the feet, lifting the leg off the floor, stretching the foot and ankle, and rotating the ankle clockwise, and then counterclockwise. The patients were asked to roll the ball by placing it between their feet and the floor during the sensory exercises and to squeeze and unclench the ball with their big toes during the strengthening exercises. Each exercise was repeated eight to ten times in the program. The exercises lasted for 10–15 min in total.

The researcher demonstrated how to do the exercises to the exercise groups face-to-face. The researcher gave the women a brochure with pictures and explanations on how to do the exercises as well as massage and stress balls to be used in the exercises. The women were told that they should do the exercise every day and should not take a break for more than 48 h. The researcher gave the women reminders about the exercises to encourage compliance with the exercises. The data were collected at two periods: week 1 (T1) and week 8 (T2).

#### Control group

The standard care protocol of the clinic (information about chemotherapy side effects) was followed for this group. No intervention was implemented for the women in the control group during the follow-up period. The data were collected at two periods: week 1 (T1) and week 8 (T2).

### Data collection tolls

Demographic and disease-related characteristics of the participants were analyzed using a Descriptive Information form. The primary outcome of the study was the neuropathy severity score assessed by the researcher. The severity of neuropathy was assessed using the NCI-CTCAE v5.0 peripheral neuropathy criteria. The secondary outcome of the study was CIPN-related quality of life. The overall quality of life of the patients was assessed using the European Organization for the Research and Treatment of Cancer Quality of Life Questionnaire (EORTC QLQ-C30), and the effect of CIPN on quality of life was assessed using the EORTC QLQ-CIPN20.

The Descriptive Information form includes demographic characteristics of the women such as age, gender, and educational background and disease-related characteristics such as chemotherapy protocol and the presence of chronic diseases.

NCI-CTCAE v5.0-Peripheral Neuropathy defines the severity of organ toxicities for patients who are receiving cancer treatment. NCI-CTCAE divides the severity of peripheral neuropathy into motor and sensory peripheral neuropathy and questions the severity of the complaints that impede the patients’ daily lives and personal care.

- Grade 1: mild; asymptomatic or a few mild symptoms; no intervention required, preventive treatment if available
- Grade 2: moderate; minimal, local, or non-invasive intervention indicated; limiting age-appropriate instrumental activities of daily living (preparing meals, shopping for groceries or clothes, using the telephone, managing money, etc.)
- Grade 3: severe or medically significant but not immediately life-threatening; hospitalization or prolongation of hospitalization indicated; limiting self-care practices (bathing, dressing and undressing, feeding, using the toilet, taking medications, etc.)
- Grade 4: life-threatening consequences; urgent intervention indicated
- Grade 5: death related to an adverse event [26]

EORTC QLQ-C30 questionnaire is widely used to assess the quality of life of cancer patients. The questionnaire consists of 30 items and three subscales: general well-being, functional difficulties (physical, role, cognitive, emotional, and social functions), and symptom management (fatigue, pain, dyspnea, nausea/vomiting, and insomnia). High scores on the functional and general well-being subscales indicate a high quality of life, while low scores indicate a poor quality of life. Low scores in the symptom management subscale indicate a high quality of life. Cankurtaran et al. conducted the Turkish validity and reliability of the questionnaire, and Cronbach’s alpha coefficient was reported to vary between 0.56 and 0.85 [27].

EORTC QLQ-CIPN 20 questionnaire was developed to determine the symptoms of CIPN and the effect of functional limitations caused by these symptoms on patients. The questionnaire has 20 items and 3 subscales: sensory (tingling, numbness, pain, imbalance when walking or standing, ability to distinguish temperature, and hearing), motor (cramps, writing, grasping small objects, weakness), and autonomic (dizziness after changing position, impaired vision, erectile dysfunction). Higher scores in these subscales indicate more symptoms and problems, while lower scores signify fewer symptoms and problems. Önsüz and Can conducted the Turkish validity and reliability study of the questionnaire and calculated Cronbach’s alpha coefficient as 0.81 [28].

### Data analysis

The Statistical Package for Social Sciences (SPSS-25) packaged software was used to analyze the data. The Kolmogorov–Smirnov test was run to determine whether the continuous variables were normally distributed or not. While repeated measures analysis of variance was carried out to determine the differences in mean scores of the scale over time and between groups, the chi-square test was applied to determine the difference between groups in terms of categorical variables. The dependent samples *t*-test with Bonferroni correction was run to determine from which group the significant difference resulted in terms of group × time interaction and the one-way analysis of variance was carried out to assess the change in the mean scores within the groups. The statistical significance level was accepted as < 0.05.

## Results

### Descriptive characteristics

A total of 84 women were included in the study. One patient who did not use the balls, another patient who was not reached during the follow-up in the massage ball group as well as one patient who was not reached during the follow-up in the stress ball group, and two patients who could not be reached during the follow-up in the control group were excluded from the study. The sample consisted of 79 women.

The mean age of the participants was 51.76 years. Most of the women were married, they were mostly high school graduates, and most of them had no chronic disease. There was no significant difference between the groups in terms of descriptive characteristics (Table 1).

**Table 1:** Descriptive characteristics of patients

Characteristics	Massage ball	Stress ball	Control	Test statistics
	*n* (%)	*n* (%)	*n* (%)	*X*^2^; *p*^a^
Marital status				
Married	21 (80.8)	22 (81.5)	20 (76.8)	0.106; 0.997
Single	5 (19.2)	5 (18.5)	6 (23.2)	
Educational status				
Literate	14 (61.5)	16 (59.3)	11 (42.3)	7,125; 0.309
Primary school	4 (15.4)	7 (25.9)	11 (42.3)	
High school	6 (23.1)	4 (148)	4 (15.4)	
Chronic disease				
Yes	16 (61.6)	13 (48.1)	14 (53.8)	2.398; 0.301
No	10 (38.4)	14 (51.9)	12 (46.2)	
	Mean ± SS	Mean ± SS	Mean ± SS	*F*; *p*^b^
Age	50.46 ± 10.81	53.37 ± 10.43	49.23 ± 10.56	1.069; 0.348
Number of chemotherapy	4.31 ± 1.30	3.85 ± 1.70	4.38 ± 3.79	2.990; 0.164

^a^A chi*-*squared test

^b^One-way Anova

### Severity of neuropathy

There was no significant difference between the groups in terms of severity of neuropathy in the pre-test (*p* > 0.05). At the end of the 8-week exercise program, there was a significant difference between the groups in terms of the severity of peripheral neuropathy in the post-test (*p* < 0.001). Women who developed grade 2 or higher peripheral neuropathy in the post-test were 57.7% in the control group, 19.2% in the massage ball group, and 1.1% in the stress ball group. After 8 weeks of exercise with both a massage ball and a stress ball, the severity of neuropathy attenuated significantly compared to the control group (Table 2).

**Table 2:** Distribution of neuropathy severity according to pre-test and post-test

Neuropathy					
Time	Group	Grade 1 *n* (%)	Grade 2 *n* (%)	Grade 3 *n* (%)	*p*^a^
Pre-test	Massage ball	24 (92.3)	2 (7.7)	0	0.161
	Stress ball	25 (92.6)	1 (3.7)	1 (3.7)	
	Control	20 (76.9)	5 (19.3)	1 (3.8)	
Post-test	Massage ball	21 (80.8)	5 (19.2)	0	** < *.0.001***
	Stress ball	24 (88.9)	3 (1.1)	0	
	Control	11 (42.3)	12 (46.2)	3 (11.5)	

^a^A chi*-*squared test

### EORTC-C30 and CIPN 20

Table 2 shows the comparison of the pre-test and post-test scores of the groups according to the subscales of the EORTC QLQ-30. There was a significant difference between the groups in terms of role function, cognitive function, emotional function, and social function. The control group scores were lower than the exercise group scores ın the post-test (*p* < 0.05). When the change in functional scores was evaluated over time, there was a significant increase in all functional scores in the stress ball group and in cognitive and emotional function scores in the massage ball group (*p* < 0.05). Time × group interaction was statistically significant for scores of physical function (*F* = 12.797; *p* < 0.001), role function (*F* = 8.758; *p* = 0.001), and cognitive function (*F* = 3.497; *p* = 0.035) subscales. The results showed that the exercise program had a significant effect on improving physical, role, and cognitive functions (Table 3).

**Table 3:** Distribution of EORTC QLQ C30 scores according to pre-test and post-test

EORTC QLQ C30	Time	Massage ball^1^	Stress ball^2^	Control ^3^	*F*; *p*^b^	*F*; *p*^c^
General well-being	Pre-test	61.21 ± 4.33	60.80 ± 4.42	58.97 ± 2.84	0.090; 0.914	12.79; 0.181
	Post-test	69.55 ± 1.59	75.61 ± 2.98	67.53 ± 2.44	4. 495; 0.050	
	*t*; *p*^a^	− 2.253; **0.033**	− 3.564; **0.001**	− 2.287; **0.031**		
Physical function	Pre-test	58.71 ± 3.36	53.33 ± 3.93	67.43 ± 3.53	5.401; **0.006** Difference:2–3	11.15;** 0.001**
	Post-test	64.10 ± 3.41	71.27 ± 3.31	58.663.43	1.907; 0.156	
	*t*; *p*^a^	− 1.753; 0.092	− 4.645; **0.001**	1.563; 0.131		
Role function	Pre-test	78.69 ± 3.51	76.54 ± 4.64	70.51 ± 5.49	1.575; 0.214	8.758;** 0.001**
	Post-test	82.20 ± 3.17	90.47 ± 3.36	72.43 ± 4.52	7.698*; ***0.001** Difference: 2–3	
	*t*; *p*^a^	1.370; 0.183	− 4.162; **0.001**	− 1.157; 0.876		
Cognitive function	Pre-test	77.56 ± 5.14	79.01 ± 3.39	77.56 ± 4.70	0.036; 0.965	3.497;** 0.035**
	Post-test	94.87 ± 1.79	91.35 ± 2.72	79.84 ± 3.14	8.059; **0.001** Difference: 1–3; 2–3	
	*t*; *p*^a^	− 3.948; **0.001**	− 4.481; **0.001**	0.775; 0.446		
Emotional function	Pre-test	72.11 ± 5.18	68.20 ± 5.30	65.70 ± 4.89	0.391; 0.678	2.253; 0.112
	Post-test	84.93 ± 2.26	94.75 ± 2.14	83.01 ± 2.59	7.350; **0.001** Difference: 1–2; 2–3	
	*t*; *p*^a^	− 2.813; **0.009**	− 5.154; **0.001**	− 4.063; **0.001**		
Social function	Pre-test	71.15 ± 6.24	80.24 ± 3.77	75.21 ± 5.21	4.390; **0.016** Difference: 2–3	2.674; 0.106
	Post-test	75.00 ± 3.23	86.29 ± 1.89	80.89 ± 3.19	4.832; **0.001** Difference: 1–2	
	*t*; *p*^a^	− 0.782; 0.442	− 4.741; **0.001**	− 1.356; 0.187		
Dyspnea	Pre-test	20.51 ± 5.86	27.16 ± 6.42	24.35 ± 5.40	0.318; 0.729	1.704; 0.498
	Post-test	8.97 ± 2.95	7.40 ± 2.71	11.53 ± 3.67	0.445; 0.643	
	*t*; *p*^a^	2.560; **0,017**	3.649; **0.01**	2.184; **0.039**		
Nausea/vomiting	Pre-test	11.53 ± 3.03	16.06 ± 3.77	13.46 ± 4.23	0.615; 0.490	1.826; 0.442
	Post-test	1.64 ± 0.64	1.61 ± 0.61	3.20 ± 1.31	1.578; 0.213	
	*t*; *p*^a^	3.305; **0.006**	4. 315; **0.001**	2.540; **0.018**		
Appetite loss	Pre-test	25.64 ± 6.74	28.39 ± 6.82	34.61 ± 7.03	0.644; 0.443	1.342; 0.267
	Post-test	2.56 ± 1.77	7.40 ± 4.10	10.51 ± 4.16	1.759; 0.179	
	*t*; *p*^a^	3.638; **0.001**	3.703;** 0.001**	3.363; **0.002**		
Insomnia	Pre-test	44.87 ± 8.85	38.27 ± 7.48	50.00 ± 7.45	0.578; 0.551	1.095; 0.909
	Post-test	16.66 ± 3.81	19.87 ± 4.29	24.35 ± 4.35	1.059; 0.352	
	*t*; *p*^a^	3.353; **0.003**	2.753; **0.011**	2.979; **0.006**		
Pain	Pre-test	26.92 ± 4.44	30.86 ± 5.08	35.89 ± 4.40	0.917; 0.404	7.587;** 0.001**
	Post-test	14.10 ± 2.56	11.72 ± 3.42	23.33 ± 3.58	7.723; **0.001** Difference: 1–3; 2–3	
	*t*; *p*^a^	3.241; **0.03**	5.112; **0.01**	3.568; **0.08**		
Fatigue	Pre-test	47.00 ± 3.86	49.38 ± 3.94	56.41 ± 3.64	1.616; 0.205	9.856;** 0.001**
	Post-test	37.17 ± 2.46	38.51 ± 3.50	55.25 ± 3.81	12.923; **0.001** Difference: 1–3; 2–3	
	*t*; *p*^a^	2.423; **0.023**	4.580; **0.001**	0.347; 0.731		
Constipation	Pre-test	25.64 ± 5.93	26.96 ± 7.02	24.35 ± 7.29	0.991; 0.10	1.214; 0.808
	Post-test	8.97 ± 2.95	12.34 ± 5.07	6.41 ± 3.21	0.586; 0.559	
	*t*; *p*^a^	2.815; **0.009**	1.991; 0.057	2.573; **0.016**		
Diarrhea	Pre-test	14.10 ± 4.95	16.04 ± 4.83	19.07 ± 5.15	0.914; 0.27	1.06; 0.984
	Post-test	1.35 ± 1.28	2.46 ± 1.71	1.35 ± 1.28	0.798; 0.227	
	*t*; *p*^a^	2.440; **0.022**	2.508; **0.019**	2.876; **0.08**		

*p*^a^, paired sample *t*-test; *p*^b^, one-way Anova; *p*^c^, repeated measures Anova

*p*-value less than 0.05 indicates statistical significance

All symptom scores except for constipation showed a statistically significant change over time within the groups (*p* < 0.05). It was found that the pain and fatigue scores of patients in both the massage ball and stress ball groups were significantly lower than those of the control group (*p* < 0.05). When the group × time interaction was analyzed, a statistically significant time-dependent difference was found between the groups for fatigue and pain symptoms (*F* = 9.856; *p* ≤ 0.001; *F* = 7.587; *p* < 0.001). The results showed that it had a significant effect on alleviating pain and fatigue symptoms (Table 2). General well-being scores did not show statistically significant changes between groups, and the group × time interaction was not significant (*F* = 1.747; *p* = 0.81) (Table 3).

There was a significant change in sensory and motor function scores between the groups in the post-test. Sensory and motor function scores were found to be lower in the massage ball group than in the control group. Motor function scores were found to be lower in the stress ball group than in the control group (*p* < 0.05). Motor symptom scores showed a statistically significant difference group × time interaction (*F* = 154.691, *p* < 0.0001) (Table 4).

**Table 4:** Distribution of EORTC QLQ-CIPN20 scores according to pre-test and post-test

EORTC QLQ-CIPN20	Pre-post test	Massage ball	Stress ball	Control	*F*; *p*^b^	*F*; *p*^c^
Sensory	Pre-test	11.68 ± 13.07	19.34 ± 19.82	13.10 ± 16.47	1.580; 0.213	5.109; 0.079
	Post-test	9.25 ± 6.21	14.50 ± 9.62	17.52 ± 14.83	3.469; **0.036** Difference: 1–3	
	*t*; *p*^a^	1.135; 0.267	1.143; 0.264	− 2.449; **0.022**		
Motor	Pre-test	13.37 ± 8.19	22.22 ± 17.16	19.78 ± 17.69	2.436; 0.094	4.287;** 0.017**
	Post-test	15.20 ± 8.73	15.11 ± 10.04	26.19 ± 14.14	6.720; **0.002** Difference: 1–3; 2–3	
	*t*; *p*^a^	− 1.03; 0.327	1.430; 0.165	− 2.697; **0.012**		
Autonomic	Pre-test	9.87 ± 5.44	5.92 ± 3.42	11.79 ± 2.95	0.973; 0.077	1.664; 0.760
	Post-test	7.69 ± 1.67	7.40 ± 11.62	12.43 ± 1.60	2.823; 0.066	
	*t*; *p*^a^	− 1.072; 0.294	1.192; 0.244	− 0.972; 0.340		

*p*^a^, paired sample *t*-test; *p*^b^, one-way Anova; *p*^c^, repeated measures Anova

*p*-value less than 0.05 indicates statistical significance

## Discussion

### Severity of neuropathy

New evaluation and care strategies for symptom management have gained importance due to the rising prevalence of cancer, the expansion of chemotherapy indications, and the development of new chemotherapeutic agents with side effects such as peripheral neuropathy and pain. A study conducted on patients with colorectal cancer reported that balance, coordination, resistance, and strength exercises were done twice a week for 8 weeks in the exercise group, and the severity of peripheral neuropathy was attenuated after exercise [29]. Another study reported that the limb exercise program significantly improved patient-reported CIPN [24]. This study evaluated the effect of exercise on the development of peripheral neuropathy in patients with breast cancer on taxane chemotherapy and reported that the severity of neuropathy decreased after an 8-week exercise with a massage or stress ball compared to the control group (*p* < 0.05). The fact that the combined exercise intervention (sensory exercises and strengthening exercises) in this study improved muscle strength and alleviated CIPN symptoms may be the reason for the decrease in neuropathy severity.

### EORTC 30 and CIPN 20

Exercises are reported to be an alternative therapy to enhance the quality of life of people with neuropathy by increasing activities of daily living and relieving pain [7]. In their study, Andersen Hammond et al. found that the neuropathic pain score declined significantly in the home-based nerve stretching exercise group compared to the control group [30]. A study by Ikio et al. reported that the reduction in activities of daily living in the exercise group after the hand exercise program was significantly less than in the control group [13]. This study also indicated that the exercise program had a significant effect on improving physical, role, and cognitive functions. The hand and foot exercises performed by patients are dynamic and functional. The fact that exercise programs help patients maintain independent functions may have contributed to an improvement in functional status.

It has been reported that hand exercises can be effective in reducing pain intensity [13]. In the study by Eroğlu and Kutlutürkan, it was found that patients experienced less pain intensity after hand-foot exercises and that peripheral neuropathy symptoms decreased compared to the control group [12]. In another study that examined the effect of exercise on the prevention of peripheral neuropathy, patients applied sensory-motor and resistance exercises, and sensory neuropathy symptoms in the feet were less common in the exercise groups compared to the control group. Also, muscle strength and quality of life were significantly better in the exercise group compared to the control group [31]. This study also revealed that the exercise program had a significant effect on alleviating the symptoms of pain and fatigue. Improvements in symptoms and increased functional mobility may have contributed to a decrease in perceived fatigue. Combined exercise practices may have relieved pain by suppressing central sensitivity to pain perception.

Exercise has a significant effect on strengthening muscular strength, improving balance, and alleviating symptoms in patients with peripheral neuropathy [32]. A study reported that neuropathy scores significantly declined in patients with peripheral neuropathy, who applied a home-based strengthening and balance exercise program for 10 weeks [33]. This study also revealed that motor symptoms were significantly reduced in the exercise group. It has been reported that using various exercise tools, such as foam rollers, roller sticks, and massage balls, creates a self-massage effect and improves flexibility without compromising muscle strength or performance [8]. However, this study revealed that there was no significant difference between exercise with a massage ball and exercise with a stress ball. Considering that exercise prevents muscle weakness caused by peripheral neuropathy and reduces motor deficits, the reduction in motor symptoms is compatible with the literature. These exercises that can be done at home enable patients to take an active role in symptom self-management and reduce the neuropathy-associated impairment in quality of life.

## Limitations

One limitation of this study is that the data were collected at a single center. Second, we re-evaluated the indicators only 8 weeks after the intervention and the long-term effect of the exercise intervention cannot be determined. Third, since the data were collected by the investigator, blinding was not possible. Finally, objective variables were not measured (fine manual dexterity and sensory-motor function, touch detection thresholds, vibration sensitivity, tendon reflexes, strength assessments, etc.), and the outcome measures were evaluated only with scales.

## Conclusion

CIPN is a dose-limiting side effect of taxane prescribed to treat breast cancer. Peripheral neuropathy is one of the most devastating symptoms experienced by the patients. It is very important to generate research-based shreds of evidence to help these patients. It is crucial to generate research-based evidence to reduce the symptoms of CIPN and help cancer patients with it. This study indicated that hand-foot exercises with a massage ball or a stress ball attenuated the severity of CIPN. This study showed that exercise can positively affect CIPN in women with breast cancer treated with neurotoxic chemotherapy and has a positive effect on symptoms such as pain and fatigue. Simple home exercises that require no special tools can be done daily in a short period of time to avoid neuropathy. Teaching these exercises to patients who will undergo neurotoxic chemotherapy before chemotherapy may help reduce the incidence and severity of neuropathy during and after chemotherapy treatment. It is recommended to conduct studies that include longer-term, follow-up evaluations of the type, duration, and intensity of exercises that are effective on CIPN.

## Data Availability

Not applicable
